# Conformational Analyses of the AHD1-UBAN Region of TNIP1 Highlight Key Amino Acids for Interaction with Ubiquitin

**DOI:** 10.3390/biom15030453

**Published:** 2025-03-20

**Authors:** Michael L. Samulevich, Liam E. Carman, Rambon Shamilov, Brian J. Aneskievich

**Affiliations:** 1 Graduate Program in Pharmacology & Toxicology, University of Connecticut, Storrs, CT 06269-3092, USA; michael.samulevich@uconn.edu (M.L.S.); liam.carman@uconn.edu (L.E.C.);; 2 Department of Pharmaceutical Sciences, School of Pharmacy, University of Connecticut, Storrs, CT 06269-3092, USA

**Keywords:** TNIP1, ubiquitin, intrinsically disordered proteins (IDPs), conformational flexibility, amino acid mutations, bioinformatics

## Abstract

Tumor necrosis factor ɑ (TNFɑ)-induced protein 3 (TNFAIP3)-interacting protein 1 (TNIP1) is genetically and functionally linked to limiting auto-immune and inflammatory responses. We have shown that TNIP1 (alias A20-binding inhibitor of NF-κB 1, ABIN1), functioning as a hub location to coordinate other proteins in repressing inflammatory signaling, aligns with biophysical traits indicative of its being an intrinsically disordered protein (IDP). IDPs move through a repertoire of three-dimensional structures rather than being in one set conformation. Here we employed bioinformatic analysis and biophysical interventions via amino acid mutations to assess and alter, respectively, conformational flexibility along a crucial region of TNIP1, encompassing the ABIN homology domain 1 and ubiquitin-binding domain in ABIN proteins and NEMO (AHD1-UBAN), by purposeful replacement of key residues. In vitro secondary structure measurements were mostly in line with, but not necessarily to the same degree as, expected results from in silico assessments. Notably, changes in single amino acids outside of the ubiquitin-binding region for gain-of-order effects had consequences along the length of the AHD1-UBAN propagating to that region. This is evidenced by differences in recognition of the partner protein polyubiquitin ≥ 28 residues away, depending on the mutation site, from the previously identified key binding site. These findings serve to demonstrate the role of conformational flexibility in protein partner recognition by TNIP1, thus identifying key amino acids likely to impact the molecular dynamics involved in TNIP1 repression of inflammatory signaling at large.

## 1. Introduction

**Figure 1:**
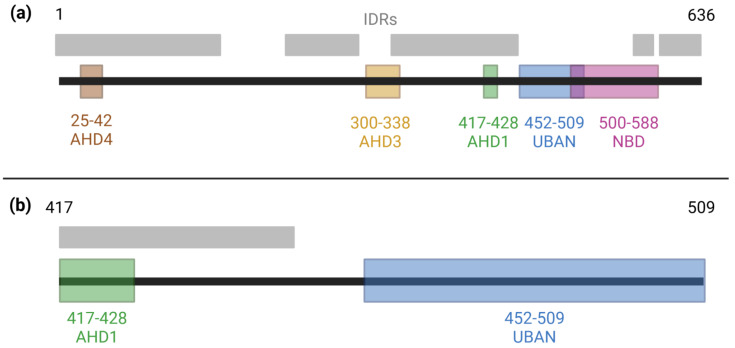
Domains and intrinsically disordered regions of TNIP1. (**a**) Known domains along the full-length TNIP1 protein (amino acids 1–636) as previously reported [29,30,31,32]. Intrinsically disordered regions spanning amino acids 1–163, 217–290, 324–450, 460–539, 563–584, and 597–636 were determined as amino acids with ≥0.5 score in the metapredictor of disorder, PONDR-FIT [33]. (**b**) A focused view of the region of the contiguous AHD1 and UBAN domains of TNIP1.

Tumor necrosis factor ɑ (TNFɑ)-induced protein 3 (TNFAIP3)-interacting protein 1 (TNIP1) is a regulator of nuclear and cytoplasmic signaling pathways [1,2,3]. The multidomain protein is named for its interaction with the ubiquitin editing enzyme TNFAIP3 (alias A20 [4]); however, TNIP1 has since been established to be a multi-partnered hub protein, here defined as a protein with ≥10 binding partners [5,6,7]. This along with its thermostability [8] are common traits of proteins containing intrinsically disordered regions (IDRs) [9,10]. Our laboratory has previously demonstrated that TNIP1 contains multiple IDRs (Figure 1a) and is thus an intrinsically disordered protein (IDP), or a protein broadly lacking definite secondary and tertiary structure and instead moving through a repertoire of conformations [5,11,12].

The regulatory role of TNIP1, particularly its repression of pro-inflammatory cytoplasmic signaling as in the nuclear factor kappa-B (NF-κB) pathway, has been demonstrated through its knockdown in cultured cells yielding heightened expression of pro-inflammatory cytokines [3,13,14,15] and explains its name alias of A20-binding inhibitor of NF-κB 1 (ABIN1). These experimental findings bolster the results of genome-wide association studies (GWAS) linking TNIP1 with several hyperinflammatory and autoimmune disorders, e.g., psoriasis [16,17], psoriatic arthritis [18], and systemic lupus erythematosus (SLE) [19], as we have reviewed [2,20]. Downregulation of TNIP1 has been experimentally linked to increased severity of phenotypic psoriasis [21], while introduction of a ubiquitin-binding attenuating aspartate-to-asparagine (D–N) variant of the TNIP1 protein into mice (D485N in mouse sequence, D472N in human sequence) results in an SLE-like disease state [22,23]. These results parallel the observation of reduced TNIP1 protein levels in the affected tissues of individuals experiencing systemic sclerosis and psoriasis [21,24]. While decreased TNIP1 protein levels are linked to inflammation-associated degradation, recent reports suggest that this phenomenon may demonstrate an emerging function of the protein as a selective autophagy receptor [25,26,27,28].

Presence of IDRs within TNIP1 can be expected to have consequences on its function, including its recognition and interaction with partner proteins (Figure 1a). Through their regional flexibility, IDRs may adopt conformations before partner binding, which facilitate a variety of protein–protein interactions (PPIs) or achieve these conformations upon partner recognition [34]. The transient nature of binding pockets formed by IDRs leads to high specificity but low affinity PPIs [35]. The ubiquitin-binding domain in ABIN proteins and NEMO (UBAN) of TNIP1 provides an example of this behavior through its interaction with polyubiquitin [36]. The specificity of this interaction is demonstrated through the strong preference for methionine-1 (M1)-linked polyubiquitin chains over lysine-63 (K63)-linked chains [37]. Similarly, the ABIN homology domain 1 (AHD1) allows for specific interaction with the ubiquitin-editing enzyme TNFAIP3 (alias A20) [29,38]. The UBAN and AHD1 domains jointly enable TNIP1 to repress inflammatory signaling despite its non-enzymatic nature, as they allow TNIP1 to recognize the polyubiquitin chains facilitating IκB kinase (IKK) complex activation while scaffolding the ubiquitin-editing A20 enzyme to repress this signal transduction [39,40]. Additionally, the AHD1 and UBAN domains are joined by a flexible linker region [5] with an average disorder score of 0.729 as determined by PONDR-FIT [33]. Meanwhile, the average disorder scores across the AHD1 and UBAN subdomains flanking it are 0.687 and 0.699, respectively. We have also previously reported [5] via DeepCoil the propensities for coiled-coil lengths across these three regions. Averaged within these regions, they are AHD1, 0.647; linker, 0.041; and UBAN, 0.750, consistent with alpha helical domain potential overlapping IRDs [41,42]. Given the intrinsic disorder of this region, we propose experimentally manipulating regional flexibility of the region encompassing the AHD1 and UBAN domains may impact its range of conformations and ultimately partner recognition.

Herein, we investigate the role of regional flexibility of the AHD1-UBAN of human TNIP1 (amino acids 417–509, Figure 1b) in its propensity towards partner-induced conformational changes, particularly as these pertain to its PPI with M1-linked triubiquitin (M1-triUb). Based on predicted residue conservation across species and contribution to conformational flexibility, amino acids were selected for point mutation into canonically order-promoting aromatic residues [43]. The variants predicted to have the greatest impact on structural stability were generated in recombinant AHD1-UBAN protein for secondary structure analysis as determined using far-ultraviolet circular dichroism (far-UV CD) and heteronuclear single quantum coherence-nuclear magnetic resonance (HSQC-NMR). These methods were also used to analyze shifts in secondary structure induced via M1-triUb binding. Finally, alterations in M1-triUb recognition by AHD1-UBAN were observed through binding affinity as determined by microscale thermophoresis (MST). Our results demonstrate the wholistic approach required when using in silico and in vitro methods while also linking the regional flexibility of the AHD1-UBAN construct with its optimal functioning in partner protein recognition.

## 2. Materials and Methods

### 2.1. In Silico Analyses of Disorder and Disorder-to-Order (D-O) Variant Design

Biocomputational analyses PONDR, PONDR-FIT, and Clustal Omega of full-length TNIP1 and select subregions were described in our previous report [5] for assessing amino acid sequence alignments and frequency as related to protein intrinsic disorder [44,45,46,47,48]. WebLogo3 [49] was used to visualize T-coffee analysis [48] of human TNIP1 sequence homology against the four published species, *Danio rerio* (zebrafish), *Ficedula albicollis* (collared flycatcher), *Taeniopygia guttata* (zebra finch), and *Xenopus tropicalis* (western clawed frog) with lowest (<54%) identity to the human protein sequence.

Amino acid substitutions to generate D-O variants were predicted using the “Mutator” program [50] derivative of the disorder predictor PONDR VL-XT [51]. The individual residues conferring the greatest D-O change were selected for engineering into the coding sequence of the recombinant protein (see Section 2.2). For comparison of glutamate to tryptophan and phenylalanine variants (E425W and E444F), which were predicated by Mutator for greatest D-O, we also generated glutamate to glutamine (E-Q) substitutions to maintain a similarly sized side chain albeit with negative to no charge character as previously described [50]. The effects of amino acid substitutions on surface electrostatics are visualized in Figure S1.

Effects of the Mutator amino acid substitutions as visualized by different disorder algorithm plots (e.g., VL-XT and PONDR-FIT) do vary in part because of additional training datasets in the case of PONDR-FIT. Additionally, PONDR-FIT is a meta-predictor of disorder built on PONDR VLXT (the sole predictor used by the Mutator program [50]), PONDR VL3, PONDR VSL2, IUPred, FoldIndex, and TopIDP to yield a higher degree of accuracy in its predictions [33,52,53].

### 2.2. Production of Variants

Inverse PCR mutagenesis was utilized to implement single amino acid changes in the recombinant AHD1-UBAN construct within a pET-28a(+) vector backbone. Primers were designed using the primer design tool from Takara Bio (https://www.takarabio.com/learning-centers/cloning/primer-design-and-other-tools accessed on 10 May 2023) (See Table S1). The variants were generated using the In-Fusion Snap Assembly kit (Takara Bio, Kusatsu, Japan) with 30 cycles of a 98 °C melting step for 10 s, a 55 °C annealing step for 15 s, and a 72 °C extension step for 5 s/kb before holding at 4 °C.

Production and size of PCR amplicons were confirmed using a 0.8% agarose 0.5× TBE gel run at 100 V for 100 min. Concurrently, 5 µL of each PCR reaction was treated with 2 µL of Cloning Enhancer (Takara Bio) for 15 min at 37 °C and 15 min at 80 °C. The resultant purified PCR product was ligated into a relaxed loop with Snap Assembly Master Mix (Takara Bio) at 50 °C for 15 min. Of the resultant circularized plasmid, 2.5 µL was used to transform Stellar *E. coli* (Takara Bio). Kanamycin-selected colonies were used to inoculate cultures, and plasmids were isolated using a Miniprep Spin Kit (Qiagen, Hilden, Germany). The recovered samples were sequenced with T7 and T7term primers (Eurofins, Luxembourg City, Luxembourg) to confirm success of mutagenesis.

#### Protein Purification and Quantification

The plasmids of confirmed samples were used to transform Rosetta2 (DE3) Competent Cells (BL-21 derivative, Novagen, Madison, WI, USA). For bacterial protein production and immobilized metal affinity chromatography (IMAC) purification protocols (for both AHD1-UBAN and M1-triUb), see our prior methods publication [26]. Additionally, size exclusion chromatography (SEC) was used to polish IMAC purified protein by concentrating the protein-containing fractions (as determined by SDS-PAGE and Pierce 660 nm Protein Assay (ThermoFisher Scientific, Waltham, MA, USA)) down to a 5 mL volume for injection onto a HiLoad Superdex 75 16/60 HP column. The column preconditioning buffer was contingent on the downstream analysis of the protein (see the analysis buffers used for far-UV CD in Section 2.3, HSQC-NMR in Section 2.4, and MST in Section 2.5). The column was run at 1 mL/min, and 2 mL fractions were collected over the course of the run. Purified and polished protein samples were quantitated in triplicate via a Pierce 660 nm Protein Assay using bovine serum albumin (BSA) standards (ThermoFisher Scientific) at concentrations of 2, 1, 0.5, 0.25, 0.125, and 0.063 mg/mL, and absorbance readings were determined using a SpectraMAX 190 Microplate Reader (Molecular Devices, San Jose, CA, USA).

### 2.3. Far-UV Circular Dichroism

A Chirascan V100 spectropolarimeter (Applied Photophysics, Leatherhead, UK) was used to collect data in a quartz cuvette with a 1 mm pathlength (Starna Cells, Atascadero, CA, USA). The data were measured in 1 nm steps with 2.5 s per point from 190 to 250 nm λ at room temperature. For greatest accuracy, a blank was measured immediately prior to each sample.

For post-CD analysis, the m° output from the CD spectrophotometer was blanked and converted to molar residue ellipticity (MRE or θ) for spectral normalization using the following equation:

$$MRE=(\frac{m{}^{\circ}}{10\;*\;l\;*\;M\;*\;n})$$

in which *l* is path length, *M* is molarity, and *n* is the number of peptide bonds (total number of amino acids—1); MRE is reported in units of [θ] or deg cm^2^ dmol^−1^ [54].

To calculate the MRE of the 1:1 protein mixture of triUb:AHD1-UBAN, the MRE calculation was modified as follows:

$${MRE}_{combined}=\frac{m{}^{\circ}}{10\;*\;l\;*\left(\frac{M_{AHD1-UBAN}+M_{triUb}}{2}\right)\;*\;(\frac{n_{AHD1-UBAN}+n_{triUb}}{2})}$$

The resultant MREs were converted into .txt files for submission into CDToolx [55]. The individual duplicate runs of each AHD1-UBAN variant were then averaged using the average function in CDToolx with the resulting files being exported in .txt format. The averaged spectra in .txt files were uploaded to DichroWeb (http://dichroweb.cryst.bbk.ac.uk/html/home.shtml accessed on 11 March 2024). In DichroWeb, the file was uploaded with Applied Photophysics formatting and analyzed using the CONTIN-LL (Provencher and Glockner Method) and dataset 7 (specialized for deconvoluting far-UV spectra between 190 and 240 nm λ) from [56] and the references therein (see Table S2). The averaged MRE values were then converted to molar extinction (ME) values for analysis in the standalone DichroIDP application, which accounts for database bias against IDPs [57]. ME was calculated as follows:

$$ME=\frac{\left[\emptyset \right]}{3298}$$

The resultant ME values were converted into .gen files (with values in descending order with regard to wavelengths) using CDToolx for submission into DichroIDP. All files were analyzed with a low wavelength cutoff of 190 nm using the IDP175 database.

For analysis without 2,2,2-trifluoroethanol (TFE) added, proteins were exchanged into 50 mM sodium phosphate (pH 8.0) buffer. Protein concentration was assessed via Pierce 660 analysis with four replicates, and the protein samples were diluted to a concentration of 10 µM. The prepared protein sample was stored in low-protein binding tubes (Eppendorf, Hamburg, Germany) overnight at 4 °C.

For analysis including 20% TFE, proteins were exchanged into 62.5 mM sodium phosphate (pH 8.0) buffer. Protein concentration was assessed as above, and protein samples were diluted to a concentration of 12.5 µM. The prepared protein sample was stored in low-protein binding tubes overnight at 4 °C. Approximately an hour before samples were read, 20% TFE was added to the sample (80 µL of TFE into 320 µL of 12.5 µM protein sample), diluting the final sample to 10 µM protein in 50 mM sodium phosphate buffer.

For analysis including 40% TFE, proteins were exchanged into 83 mM sodium phosphate (pH 8.0) buffer. Protein concentration was assessed as above, and samples were diluted to a concentration of 17 µM. The prepared protein sample was stored in low-protein binding tubes overnight at 4 °C. Approximately an hour before samples were read, 40% TFE was added to the sample (160 µL of TFE into 240 µL of 17 µM protein sample), diluting the final sample to 10 µM protein in 50 mM sodium phosphate buffer.

For production of difference spectra, each AHD1-UBAN variant was analyzed alone (at 10 µM) and at a 1:1 molar ratio with M1-triUb with each protein at 10 µM from which the spectrum of M1-triUb was later subtracted for comparisons as previously established [58,59,60]. M1-triUb was also analyzed alone at 10 µM; this was mathematically averaged with the AHD1-UBAN variant spectra to produce the average spectra one would expect if both proteins were in solution but not interacting (see Figure S2). The 1:1 molar ratio spectra were then subtracted from the mathematical average to produce difference spectra for each AHD1-UBAN variant.

### 2.4. Heteronuclear Single Quantum Coherence-Nuclear Magnetic Resonance

Protein labeled with ^15^N was expressed in minimal media containing 95 mM KH_2_PO_4_, 57 mM K_2_HPO_4_, 63 mM Na_2_HPO_4_, 13 mM K_2_SO_4_, 10 mM MgCl_2_, 0.2 mM CaCl_2_, 0.1 mM thiamine HCl, 13 mM EDTA, 0.4% glucose, 1× MEM vitamin solution (ThermoFisher Scientific), and 1× Trace Metal Mixture (ThermoFisher Scientific). Additionally, 20 mM (1 g/L) ^15^NH_4_Cl was added for ^15^N supplementation. M1-triUb was expressed as described in Section 2.2. NMR experiments were performed with a Varian Inova 600 MHz spectrometer equipped with a cryogenic probe (Agilent, Santa Clara, CA, USA). ^1^H–^15^N heteronuclear single quantum coherence (HSQC) experiments were performed to collect WT, E425W, P436W, E444F, D472N, E425Q, and E444Q both alone and with M1-triUb (1:1 molar ratio) in 20 mM sodium phosphate buffer (pH 5.8), 50 mM NaCl with 10% D_2_O at 25 °C. Data analysis was performed using programs made available in NMRBox [61]. Spectra were vertically scaled to show all observable peaks while minimizing noise levels.

### 2.5. Microscale Thermophoresis

Samples were analyzed as we previously described [26] for WT AHD1-UBAN and variant proteins. Each protein was fluorescently tagged using RED-tris-NTA 2nd generation dye, which ionically associates with the 6× -histidine tag. The tag has been included throughout our simulations and experiments and is retained in similar highly sensitive biophysical assays [5,26,62,63]. The labeled protein was allowed to incubate with a 16-step 2-fold serial dilution of M1-triUb ranging from 40 µM to 610 pM. Scans were performed in standard capillaries (NanoTemper, Munich, Germany) using the red excitation channel of a Monolith X instrument (NanoTemper) with excitation power set to 100%, MST power to 40%, and temperature to 23 °C. Values are presented as fraction bound based on MO.Affinity Analysis software v3 estimation of the bound and unbound fractions as described [64,65,66]. Plots were subsequently assessed for any potential overestimation of fraction bound, which may occur when saturation is not achieved.

## 3. Results

### 3.1. Selection of Residues and Their Predicted Gain-of-Order Following Amino Acid Mutation

**Figure 2:**
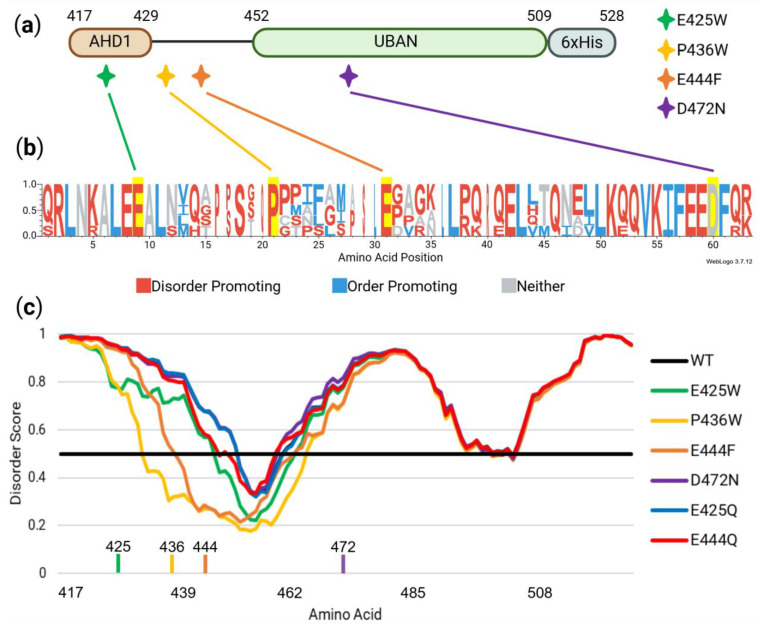
In silico assessment of potential disorder-to-order amino acid variants and sequence control variants. (**a**) Disorder-to-order sites E425W, P436W, and E444F along with an established variant, D472N, are shown in the context of their position along the AHD1-UBAN construct. (**b**) WebLogo3 visualization of T-coffee alignment analysis shows amino acid conservation present at predicted mutation sites across species with low overall TNIP1 sequence homology to human TNIP1. (**c**) PONDR-FIT analysis for predicting effects of single amino acid substitutions on regional flexibility; amino acid positions above the boundary line at 0.5 are considered to be disordered while those beneath it are ordered [33]. Positions of E425, P436, E444, and D472 are denoted along the *x*-axis. E425W, P436W, and E444F are all predicted to have a relatively large impact on regional order while D472N and control variants E425Q and E444Q cover the wildtype line with minimal deviations.

As demonstrated in our previous work [5], the amino acid sequence of the AHD1-UBAN (TNIP1^417−509^, Figure 2a) is compositionally biased away from residues typically promoting fixed protein structure (Figure 2b) [43]. Intrinsically disordered proteins (IDPs) or intrinsically disordered regions (IDRs) within proteins have a tendency towards hydrophilicity and charged amino acid content [67]. The Mutator algorithm [50] used a “replace-and-test” iterative approach, substituting every amino acid position with each of the other 19 residues and running PONDR VL-XT disorder prediction to identify substitutions with the greatest predicted order-conferring effect along the AHD1-UBAN. Disorder-to-order (D-O) substitutions highlighted via Mutator and used for eventual biophysical assays (E425W, P436W, and E444F) (Figure 2a) were selected outside the reported TNIP1 UBAN subdomain to avoid alteration of possible yet unrecognized amino acid identity-required residues for its polyubiquitin recognition function.

The established ubiquitin-binding attenuating D472N variant [22] located within the ubiquitin-binding pocket of TNIP1’s UBAN domain (Figure 2a) [37,68] was selected as a control for loss of protein function unrelated to protein conformation. This was due to its predicted lack of effect on protein secondary structure as determined by PONDR-FIT, a meta-predictor of disordered amino acid residues [33] (Figure 2c). Likewise, glutamate to glutamine (E-Q) variants were considered as controls based on predicted minimal effect on protein order/disorder (Figure 2c) and precedent [50].

The importance of the selected disorder-producing amino acid residues for mutation was further strengthened via residue conservation across distantly related species (Figure 2b). This revealed that the predicted disorder-to-order sites (along with the D472 site) are highly conserved, indicating their importance to TNIP1 functionality. The amino acids were then identified for their tendency to promote order or disorder as defined by [43]. It is notable that all amino acid sites identified for mutagenesis, save for the D472 site not predicted to impact conformational flexibility, contain residues with a tendency to promote disorder. The conservation of these disorder-promoting residues implies that amino acid identity beyond ability to promote disorder may prove crucial to AHD1-UBAN functionality.

### 3.2. In Vitro Analysis of Disorder-to-Order Variants via Circular Dichroism and HSQC-NMR

**Figure 3:**
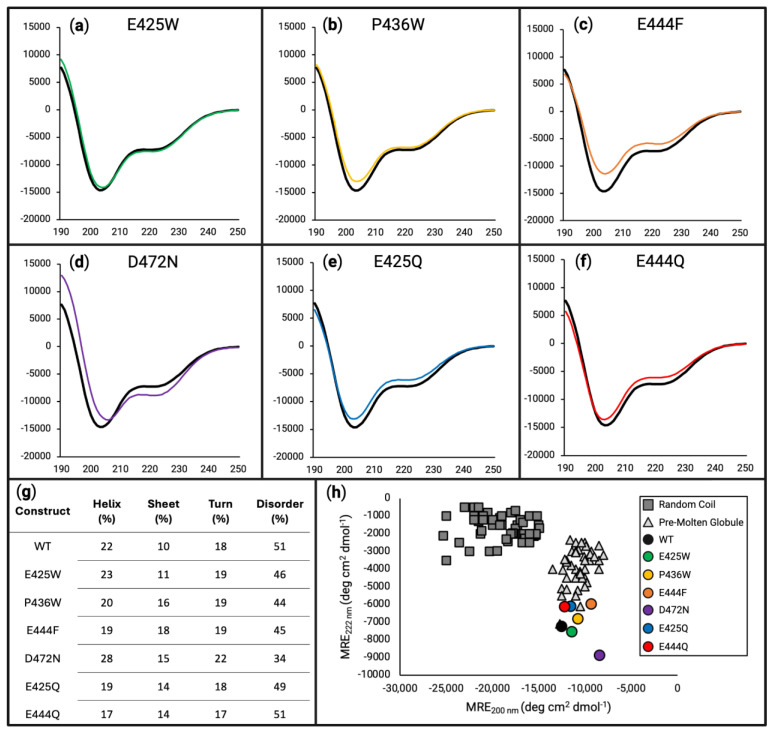
Circular dichroism estimates the effects of disorder-to-order variants on secondary structure. For panels (**a**–**f**), the x-axes are in units of wavelength (nm) while the y-axes are in units of MRE (deg cm^2^ dmol^−1^). Far-UV circular dichroism scanning from 190 to 250 nm was performed on WT (black line in panels (**a**–**f**)), E425W ((**a**), green line), P436W ((**b**), yellow line), E444F ((**c**), orange line), D472N ((**d**), purple line), E425Q ((**e**), blue line), and E444Q ((**f**), red line), each being at 10 µM in 50 mM sodium phosphate buffer (pH 8.0). (**g**) Deconvolution of data presented in panels (**a**) through (**f**) using DichroIDP. (**h**) Two-wavelength plot (222 versus 200 nm) of previously characterized random coil (dark grey squares) and pre-molten globule (light grey triangles) proteins shows all AHD1-UBAN variants grouped with the pre-molten globule cluster to varying degrees.

To investigate the potential alterations to the secondary structure of the AHD1-UBAN construct, we used far-UV circular dichroism (CD) spectroscopy. Compared with NMR, CD provides a static view, which may undersell any impact to dynamic structure. Speaking generally, an increase in the global minimum at ~200 nm, or “shallowing” of this minimum, can be interpreted as a decrease in the disorder of the protein [57,69]. When comparing the spectra of D-O and control variants of AHD1-UBAN to the WT construct (Figure 3), spectral shifts become apparent.

E425W (Figure 3a, green line) appears to have only subtle to almost no impact on the secondary structure of AHD1-UBAN. For both P436W (Figure 3b, yellow line) and E444F (Figure 3c, orange line), a shallowing of the minimum around 200 nm was observed with the change in E444F continuing along the spectrum. Notably, the effect of the E444F variant was predicted to be lesser than that of the P436W variant via PONDR-FIT (Figure 2c).

Analysis of amino acid variants based on literature precedent to provide control substitutions (glutamate-to-glutamine, E425Q, and E444Q) [50] or reported to have functional effects without characterization of secondary structural consequences (D472N) demonstrated their limitations as reference samples. Starting with D472N (Figure 3d, purple line), the left-most “shoulder” of the spectrum is increased the most drastically of all analyzed variants, demonstrating a shift towards an alpha helical character. The shallowing of the “valley” at 200 nm implies a shift from a disordered characteristic; however, the decreased ellipticity between 208 and 222 nm implies the opposite. Meanwhile, the E425Q (Figure 3e, blue line) and E444Q (Figure 3f, red line) spectra look very similar with both decreasing their initial shoulder but also increasing their 200 nm minimum and raising the spectra along its length.

To corroborate the spectral interpretations presented above, deconvolution of the data was performed using DichroIDP [57] (Figure 3g). This numerical approach captures the subtle changes between constructs, in particular showing a decrease in disorder for the anticipated D-O constructs. However, it also shows the most drastic ordering effect for D472N. Furthermore, a subtle but interesting effect on the secondary structure can be seen in E425Q and E444Q. Although the spectra of these AHD1-UBAN variants have deviated from the WT construct, their overall shifts in secondary structure do not appear to have notably increased their order. While changes in the secondary structural characteristics may be negligible, the PONDR-FIT prediction that these constructs would not impact disorder appears to hold true. Additionally, the trends of CONTIN-LL deconvolution (see Table S2) were consistent with DichroIDP results, although the newer dataset IDP175 does result in differences between the two (see Table S3).

Plotting the ellipticity at 200 versus 222 nm (adapted from [70]) (Figure 3h) is an analytical method previously proposed by Uversky et al. for distinguishing between random coil and pre-molten globule IDPs. Thinking of disorder as a spectrum, random coiled proteins are considered more disordered than pre-molten globule proteins [42]. As we previously reported [5], WT AHD1-UBAN locates amongst the pre-molten globule protein cohort. Additionally, this analysis demonstrates the different AHD1-UBAN variants also all fall within this cohort, although to varying extents; for example, D472N appears to be located away from the pre-molten globule cohort and further towards order.

**Figure 4:**
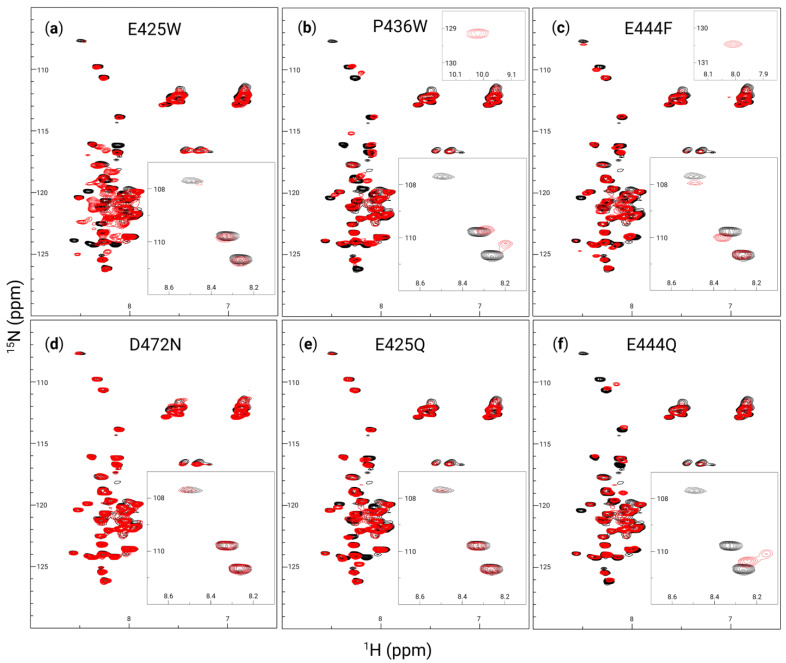
AHD1-UBAN peak shifts resulting from disorder-to-order variants. ^1^H-^15^N heteronuclear single quantum coherence (HSQC) spectra at 600 MHz collected for wildtype (black in all panels), E425W (**a**), P436W (**b**), E444F (**c**), D472N (**d**), E425Q (**e**), and E444Q (**f**) (with variants superimposed in red) at 0.3 mM in 20 mM sodium phosphate buffer (pH 5.8) and 50 mM NaCl. Insets in the upper right of panels of (**b**,**c**) show peaks unique to the spectra of P436W and E444F at (^1^H: 10 ppm, ^15^N: 129 ppm) and (^1^H: 8 ppm, ^15^N: 130.5 ppm), respectively, which fell outside of the presentation window for all other spectra. Insets in the bottom right of each panel show (^1^H: 8.1–8.7 ppm, ^15^N: 107–112 ppm).

For a more definitive view of the structural changes induced by the produced AHD1-UBAN variants, heteronuclear single quantum coherence-nuclear magnetic resonance (HSQC-NMR) was used. Compared with CD, HSQC-NMR is able to provide a more dynamic view of the protein in solution with changes in peaks representing changes in the chemical environment of amino acid residues as could be attributed to changes in conformation. The NMR spectra of WT AHD1-UBAN was used for direct comparison with all other AHD1-UBAN variants in (Figure 4a–f). It is notable that there are only about 45 peaks present in each panel despite the AHD1-UBAN construct being composed of 112 amino acids. The reduced number of visible peaks along with their sharpness implies that the most consistently flexible portion of the construct, the linker between the AHD1 and UBAN domains [71], is observable; this assumption is corroborated by the presence of the tryptophan peak in the P436W spectrum (Figure 4b, top right inset) but not in the E425W spectrum. Additionally, the amide peaks are clustered in the center (^1^H: 7.8–8.7 ppm) indicating that the majority of residues are experiencing a similar chemical environment, a characteristic of intrinsically disordered regions [72].

Despite generally poor peak dispersion, there are notable shifts between the WT AHD1-UBAN and its variants. E425W (Figure 4a) sees prominent shifts around (^1^H: 8.2 ppm, ^15^N: 117 ppm) and (^1^H: 8.3 ppm, ^15^N: 124 ppm). Particularly for the latter coordinate, it is difficult to determine where peaks shifted to. The spectrum of E425W also has broader peaks within the poorly resolved (^1^H: 7.8–8.7 ppm) peak cluster, implying some conferment of structural rigidity to these residues. While P436W (Figure 4b) sees many peak shifts in the main cluster of peaks from (^15^N: 113–127 ppm), it has very notable shifts in the upper triad of peaks from (^15^N: 108–111 ppm), even appearing to lose the top peak altogether; this peak is likely to represent a glycine residue [71]. E444F (Figure 4c) shows shifts in the seemingly sensitive (^15^N: 108–111 ppm) triad as well, although it also sees a shift near the mass of peaks at (^1^H: 7 ppm, ^15^N: 112 ppm); these peaks are commonly attributed to the amino side chain of asparagine and glutamine [73]. D472N (Figure 4d) hardly deviates from the WT spectrum, demonstrating that at least in the portion of the protein visualized here, the variant does not cause detectable changes in the secondary structure. The E425Q variant (Figure 4e) shows a lower degree of peak shifts than are seen in E425W, although some peaks in the main cluster do still shift. Inversely, the E444Q variant (Figure 4f) appears to have a greater impact on the secondary structure than E444F did; this is evidenced by the large shifts in the triad of peaks in the (^15^N: 108–111 ppm) range as well as the seeming loss of the peak at (^1^H: 8.5 ppm, ^15^N: 120 ppm) altogether. In brief, the poorly dispersed peaks yet sharp peaks of these spectra are likely representative of the most flexible protein portions, i.e., the flexible linker. While several peak shifts are apparent between the spectra, the likely glycine upper triad of peaks (^15^N: 108–111 ppm) seem particularly sensitive to conformational changes.

### 3.3. Maximum Propensity Towards Secondary Structure Is Reduced by Variants Determined by Far-UV Circular Dichroism

**Figure 5:**
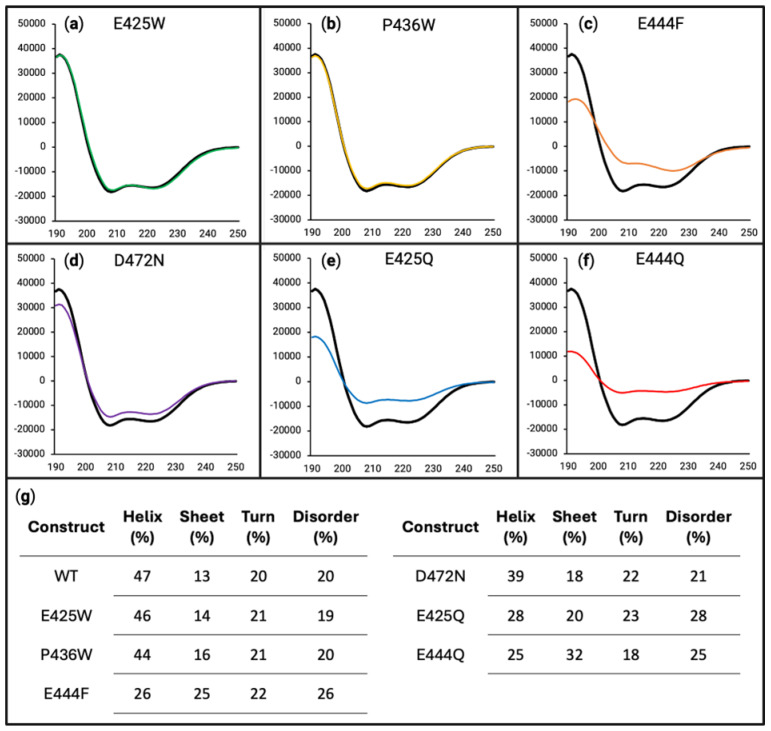
Circular dichroism of all variants in 20% 2,2,2-trifluoroethanol (TFE) (*v*/*v*). For panels (**a**–**f**), the x-axes are in units of wavelength (nm) while the y-axes are in units of MRE (deg cm^2^ dmol^−1^). Far-UV circular dichroism was performed on WT (black line in all panels), E425W ((**a**), green line), P436W ((**b**), yellow line), E444F ((**c**), orange line), D472N ((**d**), purple line), E425Q ((**e**), blue line), and E444Q ((**f**), red line), each being at 10 µM in 50 mM sodium phosphate buffer (pH 8.0) containing 20% TFE. (**g**) Deconvolution of data presented in panels (**a**) through (**f**) using DichroIDP database IDP175.

Often utilized in CD experiments, 2,2,2-trifluoroethanol (TFE) is a commonly added co-solvent used to stabilize otherwise transient alpha helices formed by a protein [74,75]. We previously established a substantial increase in secondary structure stabilization of the WT AHD1-UBAN protein in the presence of 20% TFE (*v*/*v*) and an even further stabilization of secondary structure achieved at 40% TFE (*v*/*v*) [5]. Based on these prior findings, 20% TFE was used to partially stabilize the secondary structure with the WT and D-O cohort assessed in this study (Figure 5a).

Analysis of the experimental AHD1-UBAN variants revealed that E425W (Figure 5a, green line) and P436W (Figure 5b, yellow line) had a similar propensity towards secondary structure as is seen in the WT protein. However, the potential for the secondary structure is reduced for E444F (Figure 5c, orange line), E425Q (Figure 5e, blue line), and E444Q (Figure 5f, red line), with E444Q seeming to have the greatest decrease in propensity towards secondary structure. D472N (Figure 5d, purple line) also appeared to cause a slight decrease in potential secondary structure. These trends were mirrored under a 40% TFE experimental condition (see Figure S3).

These interpretations are supported by the deconvolutions of the spectra as performed with DichroIDP (Figure 5g). For WT, E425W, and P436W, there was a similar degree of tendency towards stabilized alpha helices (47%, 46%, and 44%, respectively) at the detriment of conformational flexibility (20%, 19%, and 20%, respectively). However, E444F, E425Q, and E444Q did not decrease their disorder as much as the WT (26%, 28% and 25%, respectively, vs. 20%); this can be partially accounted for through their decreased helical characteristic, but it is also notable that they have a higher propensity towards beta sheet characteristic. In the case of D472N, there appeared to be a similar degree of lost disorder as is seen for the WT (21% vs. 20%). However, D472N had a reduced helicity (39% vs. 47%) and instead showed some of its increase in structure in its propensity towards beta sheet formation (18% vs. 13%). In sum, the majority of AHD1-UBAN variants have a diminished propensity towards alpha helical secondary structure, which is particularly notable given that both AHD1 and UBAN are alpha helical domains overlapping IDRs [41,42].

### 3.4. Partner-Induced Secondary Structure Varies Between AHD1-UBAN Variants as Determined by Far-UV Circular Dichroism and HSQC-NMR

**Figure 6:**
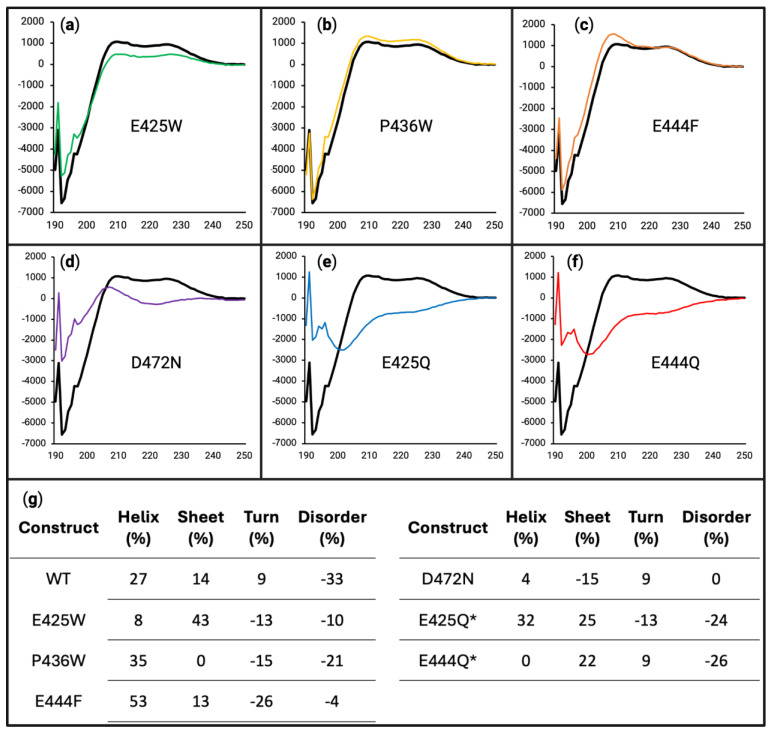
Difference spectra show induced secondary structure upon M1-linked triubiquitin binding. For panels (**a**–**f**), the x-axes are in units of wavelength (nm) while the y-axes are in units of MRE (deg cm^2^ dmol^−1^). Difference spectra were produced of WT (black line in all panels), E425W ((**a**), green line), P436W ((**b**), yellow line), E444F ((**c**), orange line), D472N ((**d**), purple line), E425Q ((**e**), blue line), and E444Q ((**f**), red line) by subtracting the mathematical average (Figure S2, dashed purple line) from the experimental 1:1 mixture of each AHD1-UBAN variant and M1-triUb (Figure S2, red line). (**g**) Percentage difference in the experimental partner-induced secondary structure versus the expected spectrum of a solution containing non-interacting AHD1-UBAN and M1-triUb. * DichroIDP was unable to deconvolute the E425Q and E444Q experimental spectra using Selcon3 analysis, so the spectra had to be scaled by 1.15× and 1.25× for analysis, respectively.

**Figure 7:**
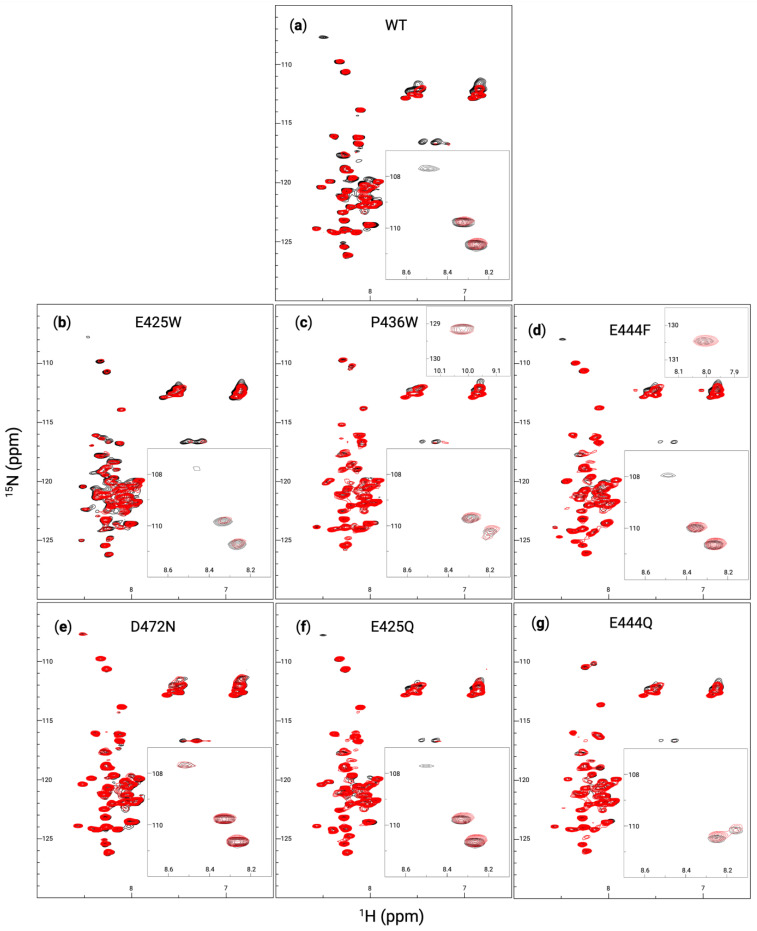
AHD1-UBAN variant interaction with M1-linked triubiquitin analyzed with NMR. ^1^H -^15^N HSQC spectra at 600 MHz collected for WT (**a**), E425W (**b**), P436W (**c**), E444F (**d**), D472N (**e**), E425Q (**f**), and E444Q (**g**) both alone (black spectra) and at a 1:1 molar ratio of 0.3 mM with M1-triUb (red spectra). Insets in the upper right of panels of (**c**,**d**) show peaks novel to the spectra of P436W and E444F at (^1^H: 10 ppm, ^15^N: 129 ppm) and (^1^H: 8 ppm, ^15^N: 130.5 ppm), respectively, which fell outside of the presentation window for all other spectra. Insets in the bottom right of each panel show (^1^H: 8.1–8.7 ppm, ^15^N: 107–112 ppm).

Although IDPs are defined by their lack of stable secondary and/or tertiary structure, secondary structure induction (especially alpha helical structure) has been widely documented to accompany partner protein binding [76,77,78,79]. Using far-UV CD and HSQC-NMR, the secondary structure of WT AHD1-UBAN and its variants were analyzed upon interaction with their partner protein M1-triUb [37,58,59] (Figure 6 and Figure 7, see pages 13 and 14).

Partner-induced conformational effects were first observed using CD as described in Section 2.3 to produce difference spectra (Figure 6). Deviations of these spectra from zero denote a secondary structure not predicted from a non-interacting mixture of proteins, thus elucidating an induced secondary structure from interaction. The WT, E425W, P436W, and E444F spectra are all relatively similar to each other in terms of shape; however, it appears that the depth of the difference is lessened in D472N, E425Q, and E444Q, showing a slightly decreased propensity for shifts in secondary structure (denoted by the large difference in the 190–210 nm range where the alpha helical shoulder typically resides). Additionally, E425W flattens more towards zero along its 210–230 nm stretch implying a slight decrease in its partner-induced turn secondary structural character. It should be noted that given the molarity of both the AHD1-UBAN variants and M1-triUb in the assay, the observed changes in secondary structure are reflective of a mixture containing both bound and free AHD1-UBAN.

An apparent difference is observed for D472N, E425Q, and E444Q, with all of these variants having less difference from the predicted non-interacting protein combination than WT and the D-O variants. For D472N, this is an expected result considering that D472N is a ubiquitin-binding attenuating variant of TNIP1. However, both E425Q and E444Q look to be interacting with M1-triUb more weakly than the WT and D-O variants. It is also possible that they are interacting with M1-triUb normally but the interaction is not inducing as large a change in secondary structure as it does in WT.

AHD1-UBAN variants and M1-triUb, each in a 1:1 molar ratio mixture, were also examined using HSQC-NMR (Figure 7). However, the minimal number of peak shifts for each variant again implies that the detectable residues are not within the UBAN domain, to which M1-triUb directly binds. The WT AHD1-UBAN spectrum (Figure 7a) aligns with prior literature [5] demonstrating some peak perturbations, particularly in the loss of the peak at (^1^H: 8.5 ppm, ^15^N: 108 ppm) and peaks within the asparagine and glutamine groups at (^1^H: 7 ppm, ^15^N: 112 ppm) and (^1^H: 7.5 ppm, ^15^N: 112 ppm) [73]. E425W (Figure 7b) and P436W (Figure 7c) also show some peak perturbation, although less than the broad rearrangement expected based on the induced structure demonstrated in CD (Figure 6). However, E425W loses its weak peak at (^1^H: 8.5 ppm, ^15^N: 108 ppm) while the spectra of P436W never showed that peak. E444F (Figure 7d) also lost its peak at (^1^H: 8.5 ppm, ^15^N: 108 ppm); additionally, there appear to be some shifts in the asparagine and glutamine groups. As expected, D472N (Figure 7e) shows nearly no shift in any peaks. E425Q (Figure 7f) loses the (^1^H: 8.5 ppm, ^15^N: 108 ppm) peak and also shows a slight perturbation of its asparagine and glutamine residues. There appears to be almost no change in E444Q (Figure 7g) save for an apparent peak shift around (^1^H: 8.25 ppm, ^15^N: 125 ppm). The consistent loss of the (^1^H: 8.5 ppm, ^15^N: 108 ppm) peak suggests that its entrance into the intermediate exchange regime is indicative of AHD1-UBAN structural shifts akin to its state upon partner recognition.

### 3.5. AHD1-UBAN Variants Show Differences in Recognition of Partner Protein M1-Linked Triubiquitin as Determined with Microscale Thermophoresis

**Figure 8:**
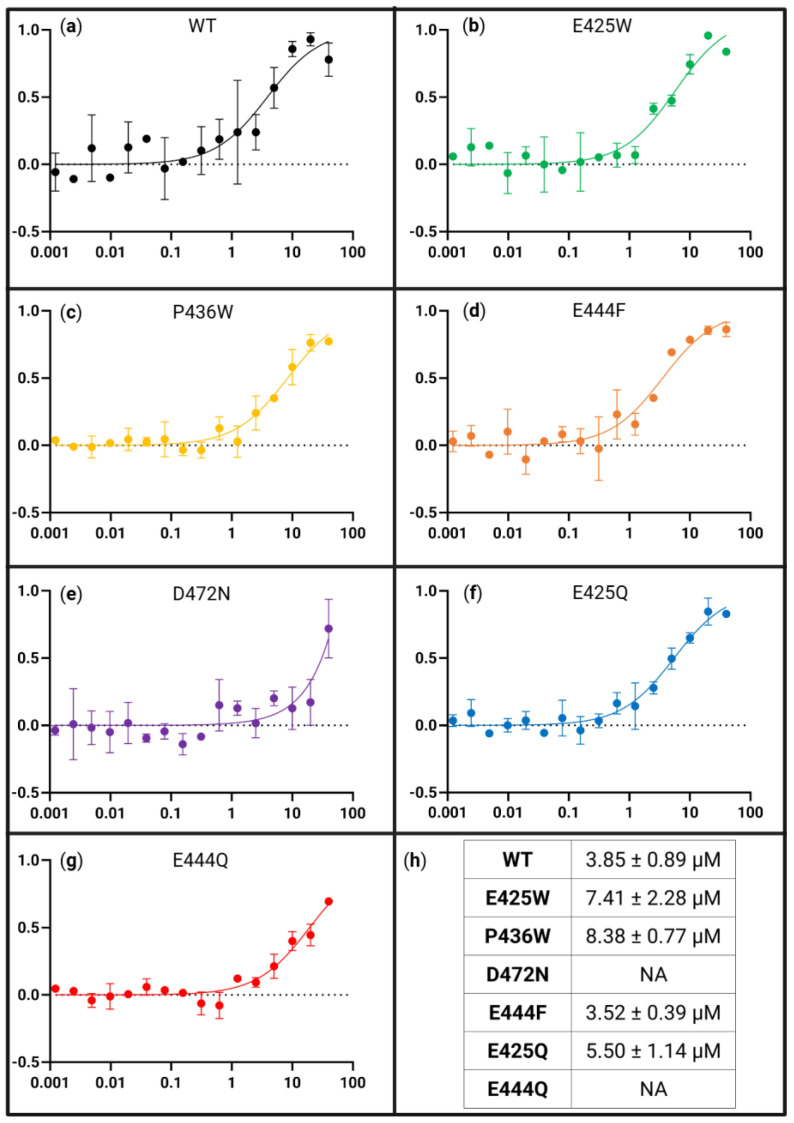
Binding affinity of all AHD1-UBAN variants for M1-linked triubiquitin as determined with MST. Panels (**a**–**g**) are presented as fraction bound (*y*-axis) versus M1-triUb concentration in µM (*x*-axis). MST curves of WT (**a**), E425W (**b**), P436W (**c**), E444F (**d**), D472N (**e**), E425Q (**f**), and E444Q (**g**). Bound fraction used for calculation was estimated by MO.Affinity Analysis software. Standard deviation of each point from n = 2 is presented. The averaged K_D_s from the datasets are presented in (**h**).

In order to quantitatively assess the impact of variations in the AHD1-UBAN sequence on partner recognition, microscale thermophoresis (MST) was utilized to determine the K_D_ of interaction for each variant across two-fold serial dilutions of M1-triUb (Figure 8). WT AHD1-UBAN showed saturation with a K_D_ of 3.85 ± 0.89 µM (Figure 8a,h); this value corroborates the low micromolar K_D_ previously reported [37,68]. D-O mutants E425W and P436W saw an approximately 2-fold increase in K_D_ (7.41 ± 2.28 µM and 8.38 ± 0.77 µM, respectively) (Figure 8b,c,h).

We compared this lessening of affinity for M1-triUB by amino acid changes outside of UBAN with the extensive reduction in M1-triUb recognition by the UBAN region-internal D472N mutation. This residue change within UBAN is recognized in the literature as a ubiquitin-binding attenuating variant [22,37,80]. Here, it results in very low, limited interaction recognized with MST assays in which only the highest M1-triUb concentration demonstrated thermophoretic motion outside of the baseline (Figure 8e). To our knowledge, this is a first-time binding affinity analysis of this interaction with the D472N variant.

The E425Q variant displayed M1-triUB binding similar to WT (Figure 8f,h) while E444Q, much like D472N, did not reach sufficient binding for a K_D_ to be calculated. Nevertheless, it appears from the lower M1-triUb concentration at which E444Q begins interacting with it as a ligand that this variant is binding with a higher affinity than the D472N ubiquitin-binding attenuating variant (Figure 8f–h).

## 4. Discussion

### 4.1. In Silico Analysis Reveals Conservation of Disorder-Contributing Residues

In the absence of fixed secondary or tertiary structures, IDPs move through an ensemble of conformations enabling interaction with a large repertoire of protein partners ([81,82] for review). Here, we present an analysis of the relative role single amino acid residues play in the regional flexibility of the AHD1-UBAN regions of the IDP TNIP1. These positions and residue replacements were identified using the Mutator program [50], which identified acidic glutamate residues E425 and E444 along with the classical “helix breaker” proline residue [83] P436 and indicated their replacement with the aromatic and hydrophobic tryptophan and phenylalanine residues, respectively, (Figure 2a) for the most predicted gain of order. Beyond their hydrophobicity, tryptophan and phenylalanine have also been demonstrated to be less frequent in the sequences of IDPs and IDRs compared to the known proteome [84]. The sites of the amino acids chosen for mutation are conserved across divergent vertebrates, humans, and the four vertebrate, non-mammalian species (zebrafish, collared flycatcher, zebra finch, and western clawed frog) aligned for (Figure 2b). This supports their functional importance, which may be through contribution to local disorder for overall conformational flexibility in innate protein structure (Figure 2c) or protein–protein interaction. Conservation of these residues for disorder within this region is present despite the otherwise apparent limited amino acid residue identity [5].

The coinciding positioning of residues predicted to confer the most D-O change with the same residue conservation across diverse species supports an emerging re-evaluation of amino acid variance in IDRs across species’ homologous proteins and protein families [85,86,87]. Specifically, disordered regions may need to retain function (i.e., conformational flexibility conferred by particular residues) as much as ordered regions preserve function from structure through conserved residue identity. This is likely of increased relevance to regions with a propensity for secondary structure, e.g., AHD1-UBAN, where specific amino acids are often more evolutionarily constrained (Figure 2b). Conservation of disorder, rather than specific amino acid identity, may account for the apparent limited percent identity typical of IDP multiple sequence alignments [82,88]. In sum, despite general expectation that disordered regions would have very limited selective pressure to retain residue identity for a particular structural conformation, intrinsically disordered regions do not a priori equate to overall loss of conserved peptide sequences [82,87]. This has led to the IDR descriptor of “constrained disorder” [87] with our data supporting that for AHD1-UBAN.

### 4.2. In Vitro Characterization of Secondary Structure Demonstrates Ordering Effect

WT AHD1-UBAN and its predicted D-O variants were subjected to secondary structure assessments in vitro using far-UV CD and HSQC-NMR. Via CD analysis, the WT AHD1-UBAN spectrum showed distinctive features of disordered proteins, e.g., the global minimum occurring at approximately 200 nm (Figure 3). The spectra of the AHD1-UBAN variants followed the same pattern, as would be expected given the relatively moderate predicted ordering effects of variant amino acid positions. Despite even the slight shift produced in the E425W spectrum, all predicted D-O variants achieved a ≥5% reduction in disordered content. However, the D472N mutation, projected to impact protein function with no detectable impact on conformational flexibility, was shown to deviate far from the WT with the most extensive decreased flexibility of any screened variant. This is further showcased in its placement on the 200 versus 222 nm ellipticity graph (adapted from [70]) (Figure 3h) in which D472N is located away from the pre-molten globule cohort towards greater stability. Intriguingly, the glutamic acid to glutamine (E-Q) substitutions, selected to maintain a similarly sized side chain, albeit varying in charge [50], also had spectra deviating from the WT. The deconvolution of these spectra reveals the PONDR-FIT prediction of these variants having no change to disorder holds true. Instead, these E-Q variants had less propensity towards formation of alpha helices with increases in their propensity towards beta sheet and turn characteristics.

While all AHD1-UBAN variants increased their helicity under 20% TFE conditions compared with the 0% TFE condition, the scale of potential helical conformation innately achievable by each variant differed dramatically. For instance, E444F, D472N, E425Q, and E444Q variants (Figure 5c–f.) demonstrated a notable decrease in the ability to form a secondary structure, namely alpha helices as determined by deconvolution (Figure 5g). The decrease in propensity towards helicity seen in D472N and E425Q is somewhat unsurprising, as these variations occur in the helical domains of UBAN and AHD1, respectively. However, E425Q has a much greater impact than E425W, the variant it was meant to control for. It could be that the altered charge from the polar Q residue substitution has a greater impact on the ability of the region to fold than the nonpolar W residue. This suggests the importance of the negative charge at the 425 site while additionally providing an explanation for the lack of effect observed in P436W.

The helical propensity of E444F and E444Q was also impacted by TFE experimental conditions. This was more surprising due to their location outside of the AHD1 and UBAN domains; however, this influence can potentially be explained by interaction between the two domains. While the crystal structure of the AHD1 domain has yet to be captured (seemingly due to its position at the end of the flexible linker in AHD1-UBAN constructs, see [37]), the structure as we have generated (see Figure S4) using AlphaFold2 predicts in its highest confidence model that the AHD1 domain folds toward the flexible linker to interact with the beginning of the UBAN domain. Therefore, if the flexible linker is disrupted, it may prevent the intramolecular interaction of the AHD1-UBAN construct, which would otherwise induce the secondary structure of the domains.

### 4.3. Partner-Induced Structural Effect Versus Loss of Conformational Flexibility

Secondary structure was also analyzed using HSQC-NMR, which upon analysis appeared to be chiefly visualizing flexible residues (i.e., those in the flexible linker) as evidenced by the appearance of a tryptophan residue in the P436W spectrum but not in the E425W spectrum (Figure 4b and 4a, respectively). However, there were peaks in a triad between (^15^N: 108 and 111 ppm) likely representing glycine residues that were sensitive to the structure induced by partner binding, particularly the upper peak of this group at (^1^H: 8.5 ppm, ^15^N: 108 ppm) [71]. This upper peak became undetectable, likely due to intermediate exchange, upon partner-induced conformation changes from M1-triUb recognition in the spectra of WT, E425W, E444F, and E425Q (Figure 7a, 7b, 7d, and 7f, respectively). Notably, P436W and E444Q innately lacked this peak (Figure 4c,e), possibly implying that this residue was already in an intermediate exchange regime for these constructs akin to its dynamics upon partner recognition. Intriguingly, the three glycine residues within AHD1-UBAN are all within the flexible linker and in close proximity to the P436 and E444 sites (G441, G445, and G447). Given this proximity, it is surprising that the chemical environment of the peak at (^1^H: 8.5 ppm, ^15^N: 108 ppm) does not extend to these peaks. Furthermore, the observation that E444F does not also induce an intermediate exchange environment for this peak implies the greater effect of introducing an acidic residue than a large, non-polar residue to this region.

Partner-induced structure was also examined with far-UV CD, demonstrating that the induction of the secondary structure is similar to the WT in the E425W, P436W, and E444F variants (Figure 6a–c). As would be expected from the reduction in ubiquitin recognition of D472N (Figure 6d), this variant shows almost no partner-induced structural shift. However, E425Q and E444Q also showed a greatly diminished secondary structure upon M1-triUb exposure. In the context of the binding affinities revealed via MST, these results align with the vastly decreased binding affinities seen in D472N and E444Q (Figure 8e,g,h). However, E425Q had a similar binding affinity to the WT, suggesting that its lack of induced secondary structure could be a result of its innate propensity towards the M1-triUb binding conformation. Further, E425W and P436W saw modest order conferral and a two-fold decrease in their binding affinity for M1-triUb. This is in contrast to a similar conferral of order seen in E444F, which shares the binding affinity of the WT. From these results, it would appear that regional flexibility can only be beneficial to the partner recognition of AHD1-UBAN.

## 5. Conclusions

We utilized in silico computational and in vitro biophysical methods to identify and assess secondary structure and PPI consequences of single amino acid substitutions on the AHD1-UBAN region of the human intrinsically disordered protein TNIP1 given its key involvement in the protein’s repression of NF-κB activation. There was general agreement between meta-platform intrinsic disorder predictors for those amino acid substitutions and results of the in vitro assays. However, the scale of D-O change predicted in those plots was not wholly manifested in the biophysical tests indicating other environmental factors (e.g., assay conditions, in silico prediction limitations) may have been in play. Some limitations of disorder predictors to mirror the extent of structural change ultimately detected via biophysical methods have been previously considered [89]. As noted [90], such shortcomings more so point to the need for purposeful inclusion of such mutational effects in the design and training sets of a predictor rather than being held against the reliability of the initial disorder prediction. Nevertheless, there was some gain of order established in all projected D-O variants. The majority of these structural changes negatively impacted the region’s interaction with M1-linked triUb, experimentally modeling the polyubiquitinated protein TNIP1 recognized for its repression of inflammatory signal progression in the cytoplasm. This suggests that conformational flexibility (intrinsic disorder) can only be beneficial towards facilitating this PPI. However, two variants break this trend: E444F, which lost conformational flexibility but retained a similar binding affinity for M1-triUb, and E444Q, which saw no decrease in conformational flexibility but a greatly reduced ability for partner protein recognition. It is notable that both of these “outlier” variants contain alterations of the amino acid identity at the E444 position, perhaps exposing a greater need to investigate entirely unstructured protein regions. Additionally, our results also show that conformational stability is at some point required for successful PPI.

Importantly, the biophysical assays revealed for the first time that D472N, literature-defined as debilitating TNIP1-polyubiquitin PPI expectedly because of its acidic, negative-to-polar, uncharged residue chemistry, saw the greatest conferral of conformational stability. Together, the designed amino acid D-O variants and this fortuitous observation suggest conservation of conformational flexibility in the region is inherent in its optimal interaction with polyubiquitin in biophysical in vitro models, a quality which likely translates to cell signaling. It is also clear that amino acid changes such as the variations at the E425 position can have an impact beyond this residue site emphasizing the need to consider consequences of any residue change in an extended lateral context. Additionally, amino acid alterations with impacts on regional flexibility (E444F) or partner-induced conformation (E425Q) may demonstrate, through their WT-analogous partner recognition capabilities, sites allowing for the stabilization of TNIP1 into conformations favoring polyubiquitin recognition. Further studies on the impacts of these variants on cellular inflammatory signaling will extend the findings beyond these biophysical assessments.

## Data Availability

The raw data supporting the conclusions of this article will be made available by the authors upon request.

## Supplementary Materials

The following supporting information can be downloaded at https://www.mdpi.com/article/10.3390/biom15030453/s1, Figure S1: Surface electrostatics of AlphaFold-predicted WT AHD1-UBAN dimer and amino acid variants; Figure S2: Estimations of induced secondary structure upon AHD1-UBAN binding of partner protein M1-linked triubiquitin via far-UV CD; Figure S3: Circular dichroism of all variants in 40% 2,2,2-trifluoroethanol (TFE) (*v*/*v*); Figure S4: AlphaFold2 prediction of WT AHD1-UBAN dimer visualized in PyMol; Table S1: Primers used for inverse PCR mutagenesis of AHD1-UBAN; Table S2: CONTIN-LL deconvolution of far-UV CD spectra; Table S3: Percent difference between CONTIN-LL and DichroIDP deconvolution results.

## Abbreviations

The following abbreviations are used in this manuscript:

IDP	Intrinsically disordered protein
IDR	Intrinsically disordered region
WT	Wildtype
D-O	Disorder-to-order
CD	Circular dichroism
HSQC	Heteronuclear single quantum coherence
NMR	Nuclear magnetic resonance
MST	Microscale thermophoresis
M1-triUb	Methionine-1-linked triubiquitin
MRE	Molar residue ellipticity
ME	Molar extinction
TFE	2,2,2-trifluoroethanol
IMAC	Immobilized metal affinity chromatography
SEC	Size exclusion chromatography
