# Supplementary material for: Conformational Analyses of the AHD1-UBAN Region of TNIP1 Highlight Key Amino Acids for Interaction with Ubiquitin
Source: Biomolecules. 2025 Mar 20;15(3):453. doi: 10.3390/biom15030453 (PMC11940065; doi:10.3390/biom15030453)
Supplement: Supplementary file 1 [file biomolecules-15-00453-s001.zip › biomolecules-3481906-supplementary.pdf]

Table S1. Primers used for inverse PCR mutagenesis of AHD1-UBAN.

Primer	Sequence
E425W FWD	5'- CCTGGAGTGGGCACTGAGCATCCAAACCCC -3'
E425W REV	5'- AGTGCCCACTCCAGGGCCTTGTTGAGC -3'
P436W FWD	5'- ATCATCTTGGCCAACAGCATTTGGGAGCCC -3'
P436W REV	5'- GTTGGCCAAGATGATGGCGGGGTTTGG -3'
E444F FWD	5'- GAGCCCATTTGGAGCAGGGGCCCTCCTA -3'
E444F REV	5'- GCTCCAAATGGGCTCCCAAATGCTGT -3'
D472N FWD	5'- CGAGGAGAACTTCCAGAGGGAGCGCAGTG -3'
D472N REV	5'- TGGAAGTTCTCCTCGAAGATCTTCACCTGCTG -3'
E425Q FWD	5'- CCTGGAGCAGGCACTGAGCATCCAAACCCC -3'
E425Q REV	5'- AGTGCCTGCTCCAGGGCCTTGTTGAGC -3'
E444Q FWD	5'- GAGCCCACAGGGAGCAGGGGCCCTCCTA -3'
E444Q REV	5'- GCTCCCTGTGGGCTCCCAAATGCTGT -3'

Table S2. CONTIN-LL deconvolution of far-UV CD spectra. All CD spectra were also deconvoluted using the classic CONTIN-LL (Provencher and Glockner Method) and dataset 7 (specialized for deconvoluting far-UV spectra between 190-240 nm λ) from [56] and the references therein.

Experiment	Construct	Helix	Strand	Turns	Unordered
**0% TFE**	WT	0.23	0.07	0.12	0.59
	E425W	0.24	0.08	0.12	0.57
	P436W	0.21	0.11	0.13	0.55
	E444F	0.20	0.13	0.13	0.54
	D472N	0.27	0.09	0.12	0.51
	E425Q	0.20	0.10	0.12	0.58
	E444Q	0.19	0.11	0.12	0.58

**20% TFE**	WT	0.533	0.03	0.129	0.307
	E425W	0.546	0.031	0.126	0.297
	P436W	0.505	0.058	0.129	0.307
	E444F	0.345	0.147	0.133	0.375
	D472N	0.441	0.101	0.139	0.318
	E425Q	0.273	0.201	0.159	0.367
	E444Q	0.192	0.256	0.167	0.385
**40% TFE**	WT	0.729	0.016	0.108	0.147
	E425W	0.702	0.019	0.113	0.167
	P436W	0.691	0.021	0.114	0.174
	E444F	0.648	0.022	0.119	0.211
	D472N	0.679	0.02	0.114	0.187
	E425Q	0.591	0.026	0.124	0.259
	E444Q	0.584	0.026	0.125	0.264

Table S3. Percent difference between CONTIN-LL and DichroIDP deconvolution results. Numbers presented are the percentage that CONTIN-LL differs from DichroIDP (e.g., for the WT 0% TFE spectrum, CONTIN-LL overestimated the disorder of the protein by 15.10% compared to DichroIDP).

Experiment	Construct	Helix	Strand	Turns	Unordered
**0% TFE**	WT	4.55%	-32.00%	-36.11%	15.10%
	E425W	4.35%	-30.91%	-37.37%	23.04%

	P436W	5.50%	-30.00%	-34.21%	25.23%
	E444F	3.68%	-26.11%	-31.58%	19.78%
	D472N	-2.14%	-40.00%	-44.09%	50.88%
	E425Q	4.74%	-26.43%	-32.22%	17.35%
	E444Q	13.53%	-24.29%	-28.82%	13.53%
**20% TFE**	WT	13.4%	-76.9%	-35.5%	53.5%
	E425W	18.7%	-77.9%	-40.0%	56.3%
	P436W	14.8%	-63.8%	-38.6%	53.5%
	E444F	32.7%	-41.2%	-39.5%	44.2%
	D472N	13.1%	-43.9%	-36.8%	51.4%
	E425Q	-2.5%	0.5%	-30.9%	31.1%
	E444Q	-23.2%	-20.0%	-7.2%	54.0%
**40% TFE**	WT	1.25%	Undefined	-28.00%	5.00%
	E425W	8.00%	Undefined	2.73%	-38.15%
	P436W	4.70%	-310.00%	14.00%	-24.35%
	E444F	1.25%	120.00%	-20.67%	0.48%
	D472N	7.78%	100.00%	-12.31%	-22.08%
	E425Q	1.90%	-48.00%	-31.11%	29.50%
	E444Q	2.46%	-56.67%	-30.56%	32.00%

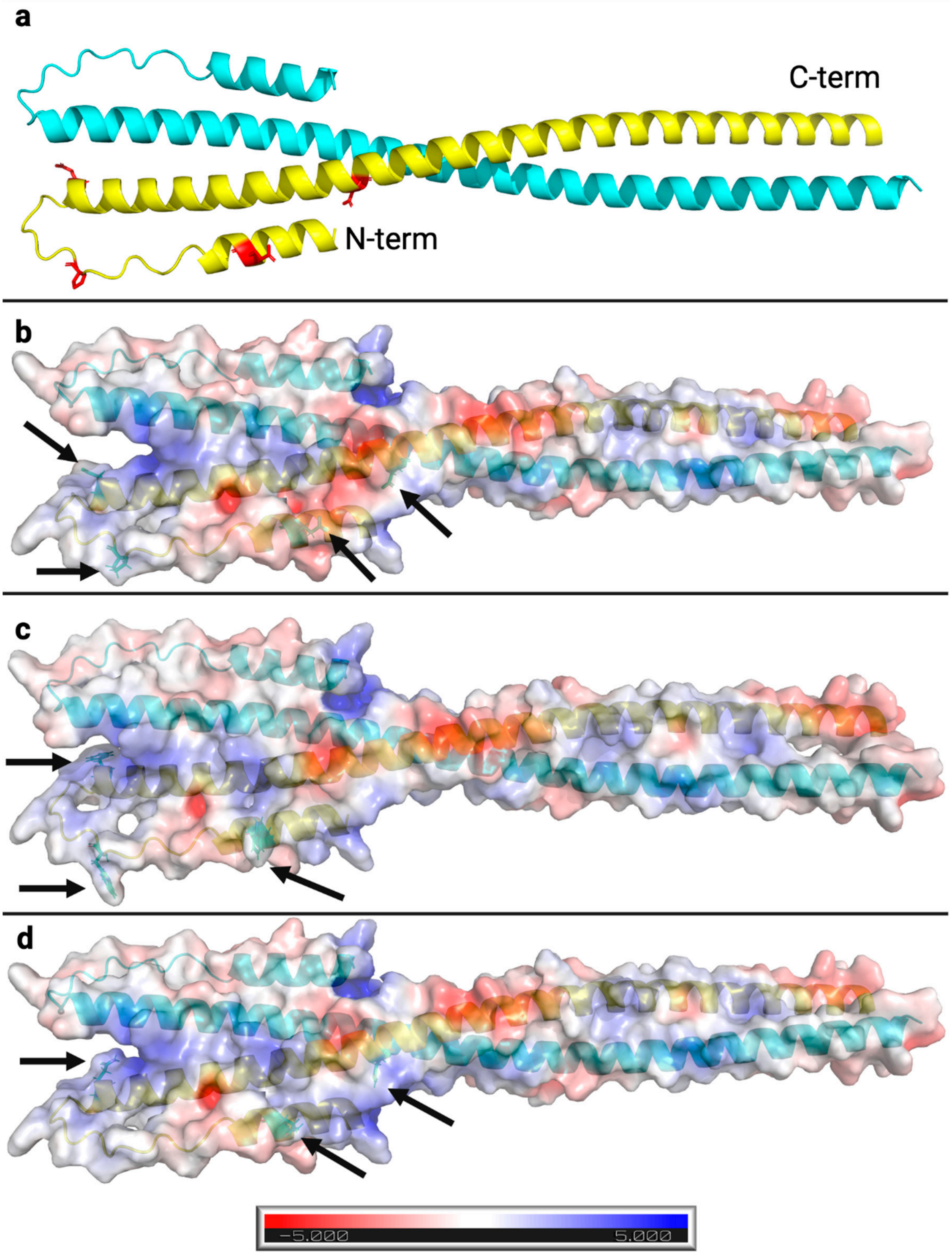
a
C-term
N-term
b
c
d
−5.000
5.000

Figure S1. Surface electrostatics of AlphaFold-predicted WT AHD1-UBAN dimer and amino acid variants. AlphaFold was used to predict the dimerized structure of AHD1-UBAN. The resulting figures were visualized in PyMol. The orientation selected was chosen to best present amino acid changes. (**a**) Dimerized AHD1-UBAN protein (yellow and cyan ribbons) with the E425, P436, E444, and D472 sites visualized as red sticks along the yellow ribbon structure. (**b**) Surface electrostatics of WT AHD1-UBAN as generated with Adaptive Poisson-Boltzmann Solver (APBS) software; E425, P436, E444, and D472 sites are visualized as lime green sticks on the yellow ribbon structure. (**c**) Following amino acid changes via the PyMol mutagenesis wizard, surface electrostatics of an AHD1-UBAN model with the predicted disorder-to-order mutations (E425W, P436W, and E444F) incorporated on the yellow ribbon structure were produced; these positions are visualized as lime green sticks. E425W appears to chiefly neutralize an otherwise acidic pocket while P436W and E444F do not look to change the neutral or basic nature of their regions, respectively. (**d**) Surface electrostatics of an AHD1-UBAN model with the predicted control mutations (E425Q, E444Q, and D472N) incorporated on the yellow ribbon structure were produced; these positions are visualized as lime green sticks. E425Q does appear to partially neutralize this otherwise acidic region, although not to the extent that E425W does. However, neither E444Q nor D472N look to alter their regional electrostatics. While not visible in this orientation, the surface impacted by D472N remains basic.

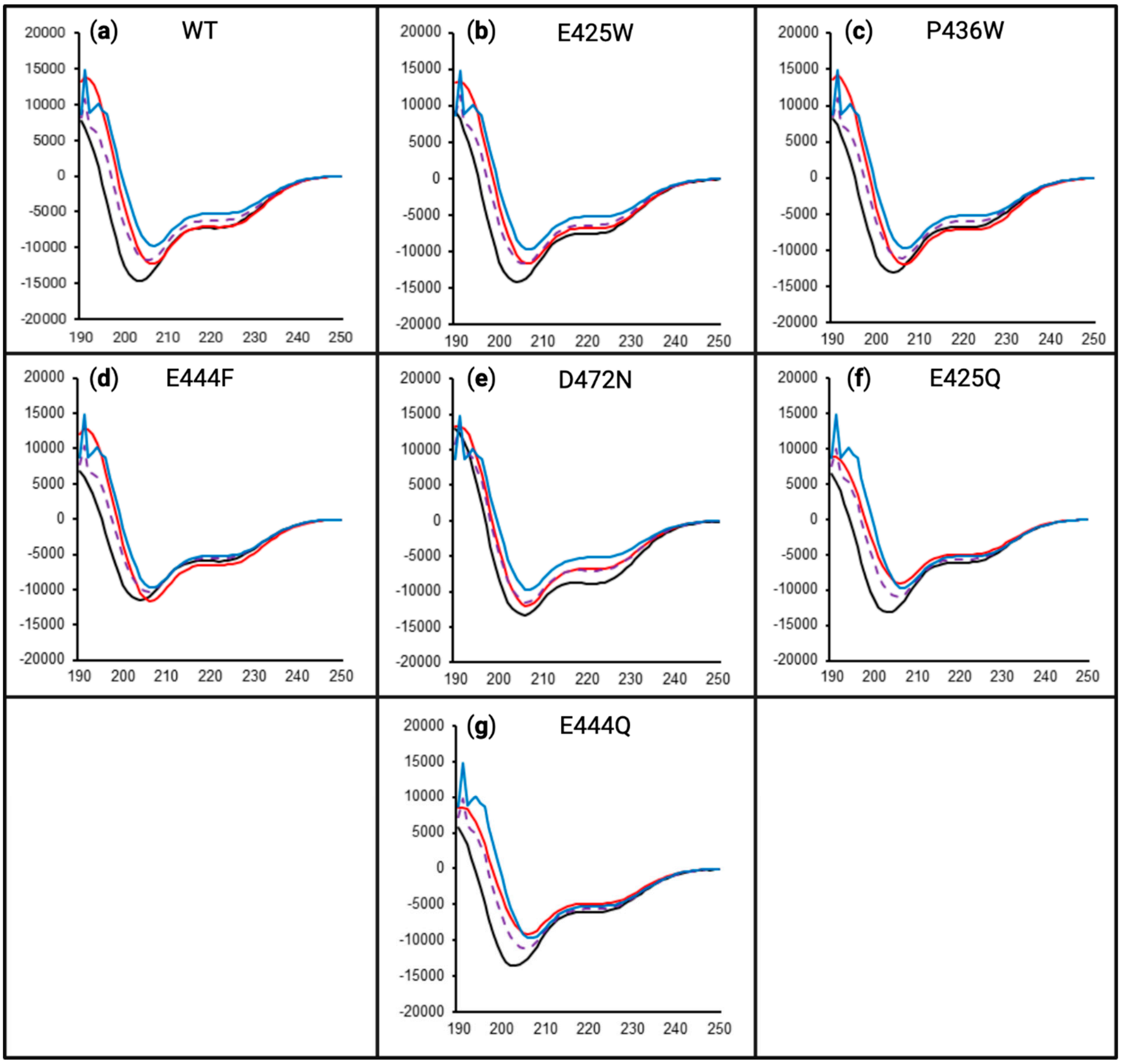

Figure S2. Estimations of induced secondary structure upon AHD1-UBAN binding of partner protein M1-linked triubiquitin via far-UV CD. X-axes are in units of wavelength (nm) while the Y-axes are in units of MRE (deg cm^2 $dmol^{-1}$). In panels (**a-g**), spectra for the AHD1-UBAN variant at 10 µM in 50 mM sodium phosphate buffer (pH 8.0) (black line) and triubiquitin at 10 µM in 50mM sodium phosphate buffer (pH 8.0) (blue line) are shown. The mathematical average of their spectra assuming a 1:1 mixture is shown by the purple dashed line, and the actual spectra resulting from their 1:1 mixture with each at 10 µM in 50mM sodium phosphate buffer (pH 8.0) with the triubiquitin spectrum subtracted is shown in red.

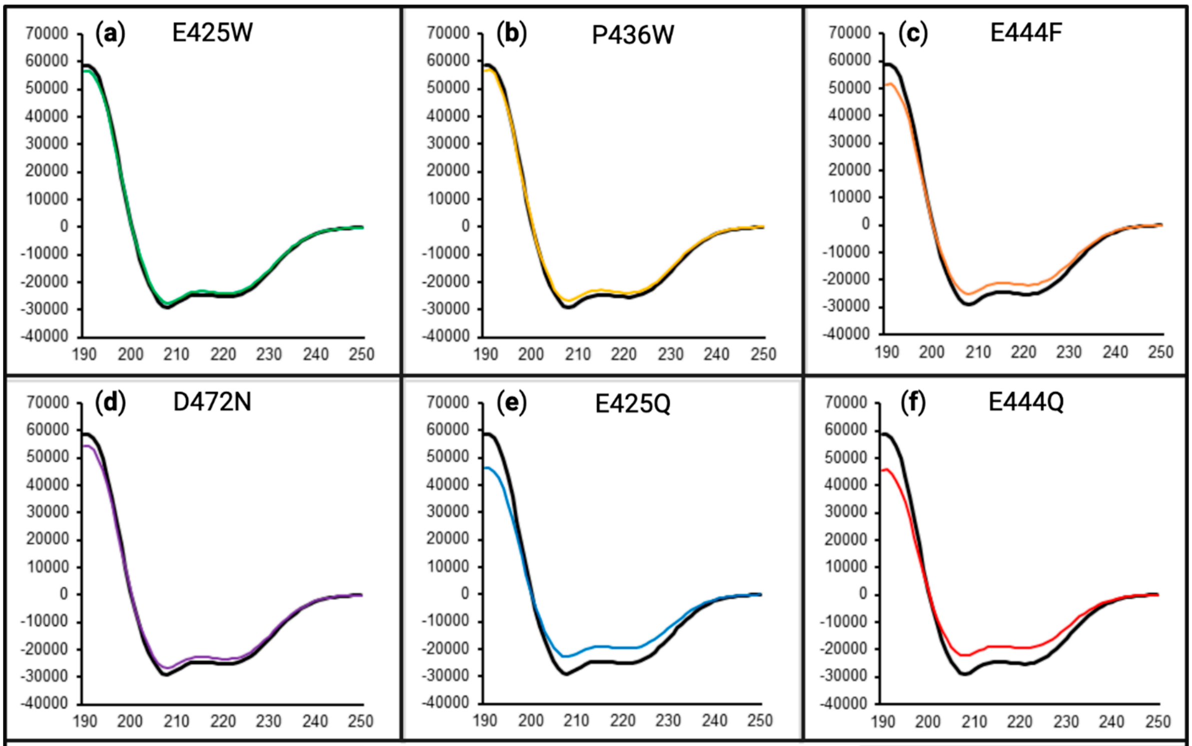

(g)

Construct	Helix (%)	Sheet (%)	Turn (%)	Disorder (%)
WT	72	0	15	14
E425W	65	0	11	27
P436W	66	-1	10	23
E444F	64	1	15	21
D472N	63	1	13	24
E425Q	58	5	18	20
E444Q	57	6	18	20

Figure S3. Circular dichroism of all variants in 40% 2,2,2-trifluoroethanol (TFE) (v/v). In panels (a-f), the X-axes are in units of wavelength (nm) while the Y-axes are in units of MRE (deg cm^{2} $dmol^{-1}$). Far-UV circular dichroism was performed on wildtype (black line), E425W (**a**, green line), P436W (**b**, yellow line), E444F (**c**, orange line), D472N (**d**, purple line), E425Q (**e**, blue line), and E444Q (**f**, red line), each being at 10 μM in 50mM sodium phosphate buffer (pH 8.0) containing 40% TFE. (**g**) Deconvolution of data presented in panels (**a**) through (**f**) using DichroIDP database IDP175.

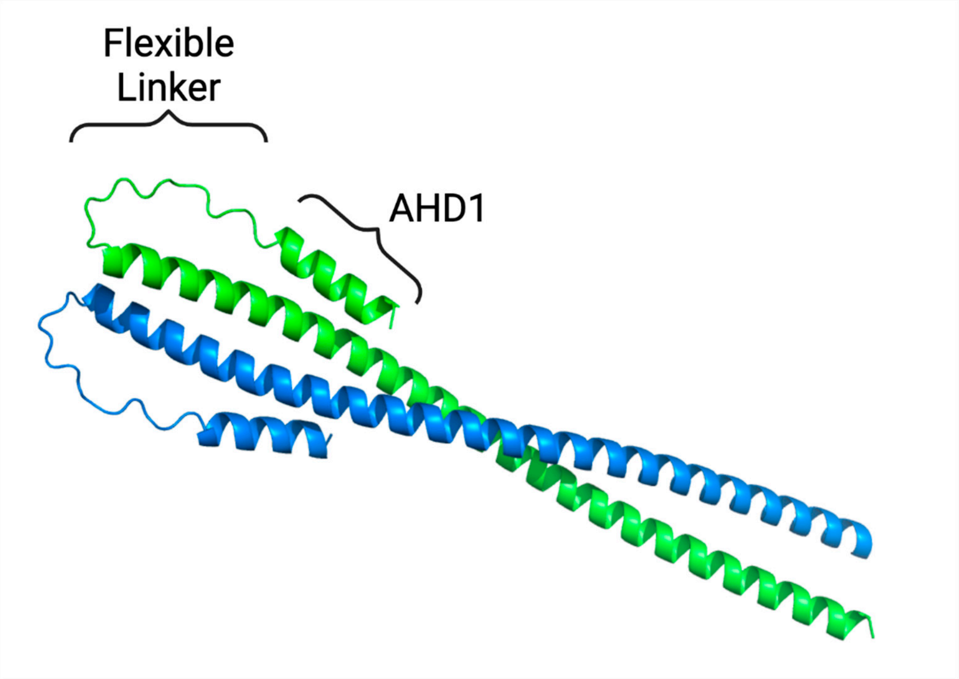

Figure S4. AlphaFold2 prediction of WT AHD1-UBAN dimer visualized in PyMol. AHD1-UBAN is shown in blue and green to differentiate between the different protein chains. AlphaFold predicts that the flexible linker domain allows for AHD1 to fold back onto the UBAN domain for self-interaction. Notably, the built-in measure of confidence predicts that the flexible linker domain structure is very low (score < 50) while the AHD1 domain is primarily low confidence (score < 70). For reference, the structure of the UBAN domain is mostly very high confidence (score > 90) with the ends being confident (score < 90).
